# Experience-based co-design of interventions to improve care quality and reduce detention under the Mental Health Act

**DOI:** 10.1136/bmjment-2026-302715

**Published:** 2026-11-04

**Authors:** Roisin Mooney, Doreen Joseph, Rose Mccabe, Karen Newbigging, Paul McCrone, Raghu Raghavan, Frank Keating, Nusrat Husain, Kamaldeep Bhui, Co-PACT Team, Tara Morey

**Affiliations:** 1 Department of Psychiatry, University of Oxford, Oxford, UK; 2 School of Health Sciences, City University, London, UK; 3 University of Birmingham - Edgbaston Campus, Birmingham, UK; 4 Greenwich Campus, London, UK; 5 De Montfort University, Leicester, UK; 6 Law and Criminology, Royal Holloway University of London, Egham, UK; 7 The University of Manchester, Manchester, UK; 8 Nuffield Department of Primary Care Health Sciences, University of Oxford, Oxford, UK

**Keywords:** Mental Health, Mental Health Services, Psychiatry

## Abstract

**Background:**

The use of compulsory admission and treatment (CAT) for mental health in England is increasing and disproportionately impacts racialised populations.

**Objective:**

This work convened stakeholders in a co-design process informed by lived experience of CAT with the aim of developing implementable recommendations intended to improve mental healthcare and reduce racial disparities.

**Method:**

Co-design workshops were attended by 96 key stakeholders in six National Healthcare Service (NHS) Trusts. Stakeholders included psychiatrists, NHS executives, service users, carers, representatives from voluntary organisations, police, nurses, occupational therapists and social workers. Touchpoints were identified from experiential data gathered through photovoice workshops and action plans formed around them. Local workshops were followed by a national knowledge exchange workshop to connect sites and consider both local and national recommendations.

**Findings:**

The recommendations included the five following quality improvement and care standards: (1) better connections between local services for preventative care, (2) build capacity among the workforce, (3) co-design trauma-informed standards for mental health inpatient care, (4) provide talking therapy before, during and after detention and (5) personalise discharge and community support plans. The need for the resources was supported by the sites, with implementation responsibilities placed in different groups in each Trust.

**Conclusion:**

People from across the systems involved in the delivery of mental healthcare, including patients and carers, have highlighted opportunities and mechanisms to prevent detention and improve the quality and safety of care.

**Clinical implications:**

We present five policy recommendations that could inform quality improvement evaluations in local systems in relation to national systems frameworks such as the Patient Carer Race Equality Framework.

**WHAT IS ALREADY KNOWN ON THIS TOPIC**

- Compulsory admission and treatment (CAT) for poor mental health disproportionately affects people from racialised backgrounds. It is unclear how to address or reduce these inequalities. This study was conducted to inform the revision of the 2008 Mental Health Act.

**WHAT THIS STUDY ADDS**

- This study developed five policy recommendations to reduce ethnic inequalities in CAT that have been developed with a diverse group of stakeholders across the healthcare system, alongside patients and their families and communities. Previous recommendations have not always accounted for such a diverse range of views from across the system. The voices of people from racialised backgrounds are often missing from policy and research conversations.

**HOW THIS STUDY MIGHT AFFECT RESEARCH, PRACTICE OR POLICY**

- This study used creative methods to represent the lived experience perspectives in co-design meetings, and facilitate participants to engage with the challenges and consider solutions taking account of multiple perspectives: patients and healthcare professionals. This study has implications for both inclusive research methods and policy.

## Introduction

 The number of people experiencing compulsory admission and treatment (CAT) for their mental health is increasing.^1^ Addressing this warrants recognition as a global priority.^2^ Receiving care in a coercive manner is known to be distressing, traumatic^3^ and in conflict with human rights-based mental health care.^4^ Data from Europe, Australia and New Zealand show that countries with a higher proportion of foreign-born people had a higher annual incidence of CAT.^1^ In England, people from racialised populations disproportionately experience CAT.^5^ In England, black people, as a subset of racialised populations, were reported to be nearly four times as likely as white people to be detained, between 1 April 2024 and 31 March 2025.

The explanations for these disparities are complex, and made more so due to much geographical variation in both the drivers of inequalities and provision of care, as well as the gap between legislative intentions and actual practice.^6^ For example, involuntary care may be used as a default or way to manage behaviour rather than as a last resort, which contradicts legal mandates for the least restrictive treatment.^6^ An international systematic review found that almost half of the literature around ethnic inequalities in detention rates offered no explanation for variation by ethnicity. The most common hypotheses include: increased prevalence of psychosis (although this should not by default result in increased coercive care), increased perceived risk of violence, increased police contact and absence or mistrust of general practitioners.^5^ There is equally little consistent evidence of what works with regard to reducing ethnic inequalities in CAT over time.

There has been little progress or change in addressing ethnic inequalities in CAT over the last 20 years^7^ which is exacerbated by insufficient attention to the drivers of CAT and of ethnic differences in CAT for people who are not routinely involved in research, yet are more likely to be detained. Many people facing multiple intersectional disadvantages are overlooked if research is overly focused on a specific demographic such as age, gender or ethnicity. Furthermore negative experiences of people with mental illness are dismissed as lacking credibility or as symptoms of their illness.^8 9^

Experience based co-design (EBCD) is a form of participatory action research that aims to improve healthcare practices and services by enabling service users, carers and staff (ground-level and management) to share their lived experiences and then collaborate and design better services.^10^ EBCD seeks to address epistemic injustice (where someone’s experiences are given less credit due to their social position) and reduce power asymmetries in research and in practice.^8^ EBCD in local mental health systems has been used to develop priority action plans tailored to the local contexts,^11^ engage diverse groups of people and develop innovative solutions to complex issues, reformulate inpatient care to better respond to power imbalances, therapeutic cynicism and time pressures.^12^ In response to these challenges, we present the outputs from one work package within a National Institute of Health and Social Care Research Policy Research Programme funded study called Co-PACT,^13^ where we applied EBCD to improve practice, policy and legislation. The broader Co-PACT study used photovoice workshops to gather lived experience data, the results of which are separately published. Information from the photovoice workshops was shared to represent the lived experience of the intended beneficiaries during the co-design workshops which are reported in this paper.

### Aims

Using lived experience data gathered using photovoice workshops from people who had experienced CAT in the preceding 2 years (that has already been published),^9 14^ we organised a series of EBCD workshops to evolve recommendations for future practice, policy and legislation.

Specifically, we sought to develop local action plans and national recommendations to reduce ethnic inequalities in CAT in collaboration with a diverse range of stakeholders and understand implementation challenges faced by EBCD participants.

## Methods

### Design and setting

EBCD is an approach that incorporates a safe space for knowledge sharing and synthesis which has been found to be an effective facilitator of knowledge mobilisation.^15^ The British Design Council Double Diamond Model for EBCD was adopted for this study. EBCD workshops were conducted in six local healthcare systems centred around NHS mental health trusts, where photovoice workshops as part of the broader Co-PACT research programme had taken place.^9^ Photovoice workshops resulted in local lived experience data in the form of images and narratives that were presented within the co-design workshops. This work has been reported separately and is already published.^9 14^

A Patient and Public Involvement Research Group (PPIRG) for the broader Co-PACT research programme consisting of six people with experience of CAT advised on how to represent the large volume of lived experience data in the co-design workshops. The preliminary experience data from the photovoice workshops that were presented in the co-design meetings are summarised in the online supplemental material (see also references to previous work^8 9 13 14^). The PPIRG also advised how we held the co-design workshops. Those with lived experience of CAT were contacted before each workshop to share with them the process and ensure that they were happy to hear the information being discussed. They were also contacted after each workshop to check that they were not distressed by the conversations about difficult experiences shared during the workshops. It was important to the research team to recognise the differential in power balances; people who had been detained against their will were in the same room as professionals who had been involved in their care and possibly CAT. For this reason, workshops were held away from NHS buildings and instead in creative community spaces.

### Participants and recruitment

Recruitment was purposeful to ensure representation by age, gender, ethnicity and lived experience as a patient or carer and by a range of relevant professionals. Critically we sought to ensure that there was not a dominant group and that the voices of lived experience were central to all discussions. We aimed to recruit 12 people per workshop to ensure a diverse range of perspectives while facilitating all participants to engage meaningfully. Mixed stakeholder workshops were conducted (as opposed to smaller groups of different types of professional) to ensure that the co-design process addressed any tensions and system barriers, as well as motivate those in attendance to progress the actions having heard different perspectives directly. At this stage it was important to secure engagement from those implementing resulting actions and those in positions of influence.

Potential participants were identified and approached by local principal investigators (PIs) in each NHS trust and local community. Every participant was given an information sheet in advance of the workshop, with the opportunity to ask questions of both the local research team and the broader university study team. Participants were asked to provide consent when attending the workshops, at the first break, after the purpose of the workshops had been presented.

### Conducting co-design workshops

Our intention was to run a series of three half-day (3–4 hours) workshops with appropriate breaks in each area over a period of 3 months.^13^ From working in each of the local systems to recruit participants for photovoice it became evident that it would not be feasible to get the right combination of people who have the ability to implement decisions made, as well as people with relevant lived experience in the room together on three separate occasions for a substantial amount of time. The study took part during the phase of recovery after the COVID-19 pandemic and there remained concern about gatherings and safe venues for convening meetings. At the time, there were additional clinical pressures due to the junior doctor strikes.

Following consultation with the independent study advisory board, we offered a more flexible approach to ensure that a diverse range of experiences were represented in the room. In four local systems we held two half-day workshops (Birmingham, East London, Derby, Oxford), and in the other two areas (Manchester, Bradford) these were condensed into a single day as it was not possible owing to capacity of those who had agreed to attend to hold two separate shorter meetings.

Workshops were facilitated by the lead author and co-PI for the study, supported by the coinvestigator team which included experts in co-design. There were two key objectives in the workshops—establishing touchpoints (opportunities to intervene and change outcomes) and developing action plans to address the established touchpoints. To begin, the research team shared a brief PowerPoint presentation around the ethos of the project, the current context with regard to the Mental Health Act (MHA) legislation and existing inequalities, who had contributed to the experience data, local examples of experience data (images with captions) and what we hoped to achieve during the workshop. Presenting different perspectives (staff and patients) was critical to getting everyone in the room to work collaboratively and avoid assigning blame for the challenges being discussed. There was no formal method for reaching consensus; rather, consensus was sought through discussion. Summary tables containing quotes from photovoice workshops where people with lived experience of CAT had discussed their experiences with each other were then shared, and participants worked in smaller groups according to which table they sat at to identify which elements of the summary table they felt were of the highest priority.

Once touchpoints had been agreed on, participants collectively began to identify opportunities for intervention and developed an action plan using the following headings: Issue, Action, Steps, Who, Time, Cost, Aim and Evaluation. All workshops were facilitated by RMo, who took advice from investigators, local stakeholders and the PPIRG effective delivery of co-design workshops.

### Documenting and following up workshops

All workshops were audio recorded and transcribed verbatim, then imported into MAXQDA. The outputs from the workshops are summarised highlighting touchpoints (opportunities for intervention to change a person’s trajectory, identified in the first co-design workshop) and co-designed action plans (developed in the second workshop). No structured qualitative analysis was conducted on the transcripts, as this was not the aim of the research, which was to develop local action plans which informed national recommendations. Data from the co-design workshops was summarised by RM, and shared with sites for feedback and iteration. The policy recommendations were developed following a knowledge exchange event which was attended by researchers, people with lived experience and representatives from each of the sites, where the co-designed action plans were presented by each of the sites. The policy recommendations were then presented to and iterated by the PPIRG and coinvestigators.

The research team followed up with those who had organised the workshops in the local systems 3–6 months after the workshops by email or at the knowledge exchange conference that was organised as part of the broader programme to understand the impact of the workshops and how action plans were being delivered. This was not an active implementation study; rather, those involved in the co-design were encouraged to make use of resources in their local systems. Participatory processes are designed to engage, empower and equip participants to take action beyond their direct participation in the study.^16^

## Results

In total 96 people attended at least one co-design workshop across six NHS trusts (see table 1 for participant characteristics).

**Table 1:** Participant characteristics

Venue	Birmingham	London	Oxford	Derby	Bradford	Manchester	Total
Workshop 1	14	18	12	11	15	14	84
Workshop 2	13	12	11	23	0	0	59
Total number of people who attended across both workshops							
	14	18	12	23	15	14	96
Characteristics of participants							
Lived experience of MHA detention	2	5	4	5	2	0	18
Informal carer	1	0	0	1	1	1	4
Psychiatrist	1	3	2	2	2	3	13
Psychologist	1	1	0	0	1	1	4
Occupational therapist	1	1	1	2	1	2	8
Allied mental health professional	0	2	1	3	2	2	10
Non-government organisation	1	1	1	2	0	0	5
Mental health nurse	2	1	1	2	0	2	8
Police	0	1	0	2	2	0	5
Judge	0	0	0	0	2	0	2
Senior NHS executives (eg, clinical director, chief medical officer, service lead)	3	2	1	3	1	2	12
Researcher	2	1	1	2	0	1	7
Sex							
Male (%)	6 (41)	10 (55)	9 (75)	13 (56)	5 (33)	8 (57)	51 (53)
Racialised populations							
Non-white (%)	11 (79)	12 (66)	2 (16)	8 (34)	9 (60)	9 (64)	51 (53)

Some participants attended both workshop 1 and workshop 2, other participants only attended one of the two workshops. This is why the total number of people who attended across both workshops, is not simply the sum of those who attended workshop 1 and those who attended workshop 2.

MHA, Mental Health Act; NHS, National Health Service.

### Key issues across sites

The table below (table 2) details the key issues that resonated across the different co-design meetings, the touchpoints that illustrated these issues and the co-designed interventions. Critically how people communicate with each other and acknowledge a person’s humanity, as well as the fact that anyone can develop poor mental health were central tenets in many proposed interventions.

**Table 2:** National priorities and action plans

Issue	Touchpoints from photovoice data and experiences shared during co-design workshops	Potential intervention(s) from co-design workshops	Agreed co-designed recommendations
Public understanding of what poor MH is and what being on an MH ward is like	Long term implications on employability. Public nature of being detained. Police engagement in detention processes.	**Public campaign—**emphasis on the variety and nature of detentions. People with lived experience wanted others to know that poor MH can happen to anyone. Improved awareness around ethnic inequalities in MH.	**CONNECT** local services to raise awareness of what poor MH looks like for both those within the MH care system and the public. **GROW** capacity to facilitate better knowledge sharing between different parts of the system as well as friends and family and prioritising the voice of the patient (particularly regarding relapse).
Poor access to services and treatment	Too complex to receive help. Not unwell enough to receive help. Discharged from hospital and on a 6-week wait list for psychological input. Waiting on a referral from a GP for over 2 weeks and sectioned while waiting.	Better signposting across different NHS and community resources for both staff and patients. **Local mapping**/consortia of available resources? (Draw on the Derby health inequalities partnership and community action model.) **Access to advocates in the community** predetention rather than once detained.	
Inconsistent transactional communication	Lack of a consistent care coordinator. Feeling ignored by nurses on inpatient units. Lack of occupational therapy on weekends. Disempowering ward rounds.	Implement constant meaningful feedback loops with accountable actions. **Staff training** that addresses the following questions: how to humanise experiences? Where is the flexibility? What does reasonable risk taking look like? **Opt out system for advocates** for inpatient wards.	**CO-DESIGN** new standards that ensure; frequency of communication, documentation that represents patient experiences and access to advocacy, reinforced with staff training.
Detention as a traumatic experience	Feeling kidnapped when being sectioned (use of black vans, poor identification of venue or staff). Likening being injected with medication to rape. Using metaphors around zoos and prisons to describe the ward environment. Lack of hospital placements available locally.	Orientating people to different situations—taking time to explain. Debrief with staff and patients after challenging situations (not only those directly involved but also observers). Review of dehumanising processes (ie, queuing up to receive medication). **Peer support or debrief when discharged** (consider model akin to birth after thoughts for new mothers).	**PROVIDE** access to appropriate therapy before during and after detention, this could be in the form of peer support or more formal therapeutic sessions.
High rates of redetention	Reliance on friends for support when in crisis and not needing to go into hospital. People reported a self-awareness of their MH deteriorating and needing help.	**Engaging family and friends** in care from the first point of contact in a meaningful and collaborative way, including access to support for them. **Implementing meaningful advanced agreements**—what’s important to me?	**PERSONALISE** discharge plans so that they reflect the individual rather than the resources available in the system.

GP, general practitioner; MH, mental health; NHS, National Health Service.

### Local touchpoints and action plans

There were multiple touchpoints (figure 1), on how poor communication was exacerbated by staff shortages and lack of capacity in health systems, as well as challenging environments due to reliance on crisis care. Quotes from the co-design meetings that relate to these touchpoints are provided in online supplemental material table 1.

**Figure 1:**
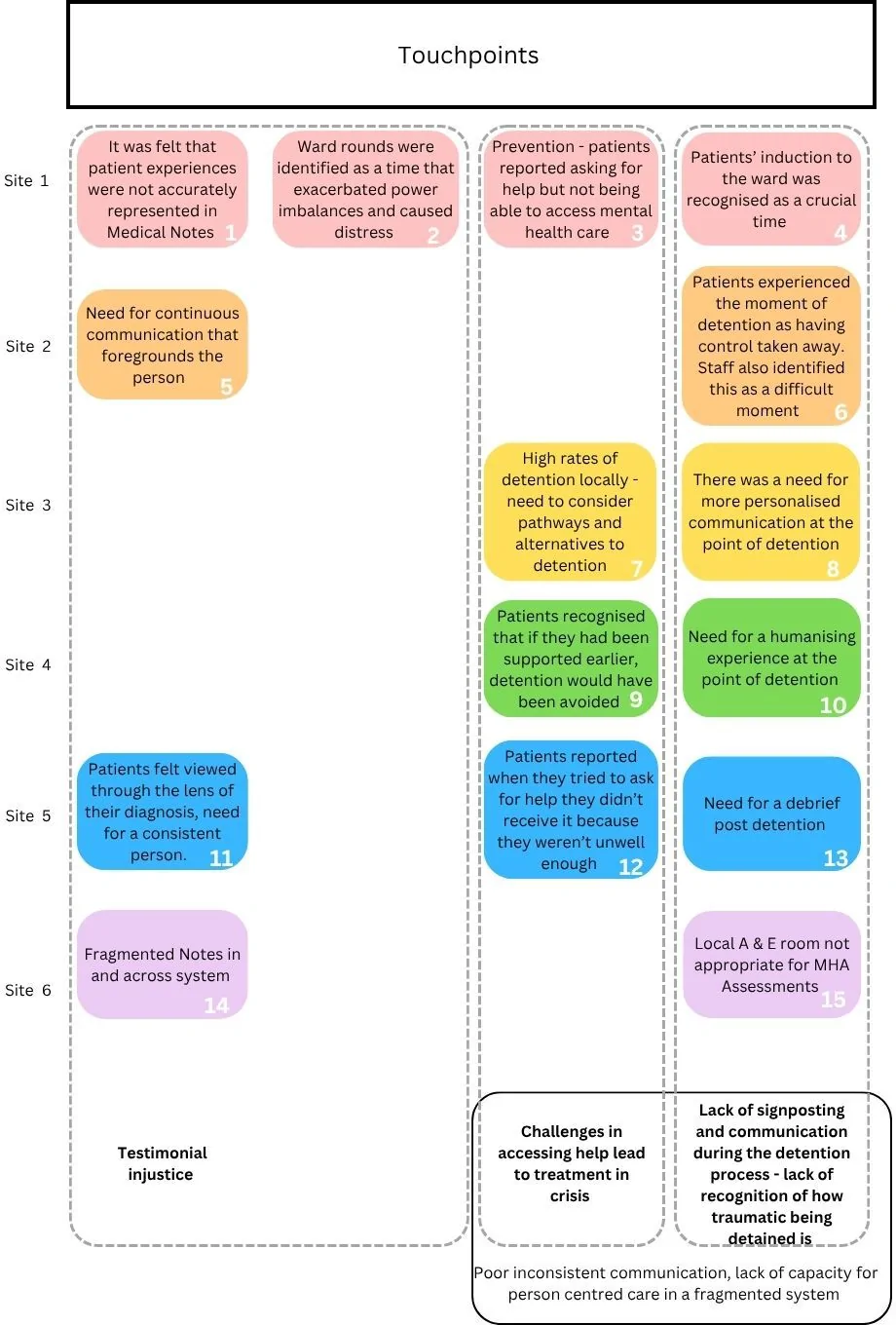
Established touchpoints (numbers in boxes index quotes in the online supplemental material table 1). A&E, accident and emergency; MHA, Mental Health Act.

### Developing action plans

The below figure (figure 2) represents the action plans that were developed in response to established touchpoints. The numbers in the figure below relate to the numbers in the table below which highlight some of the pathways and barriers to implementation that were discussed. Key points around the pathways to sustainable implementation and associated barriers that were shared in the co-design meetings are shared in online supplemental material table 2.

**Figure 2:**
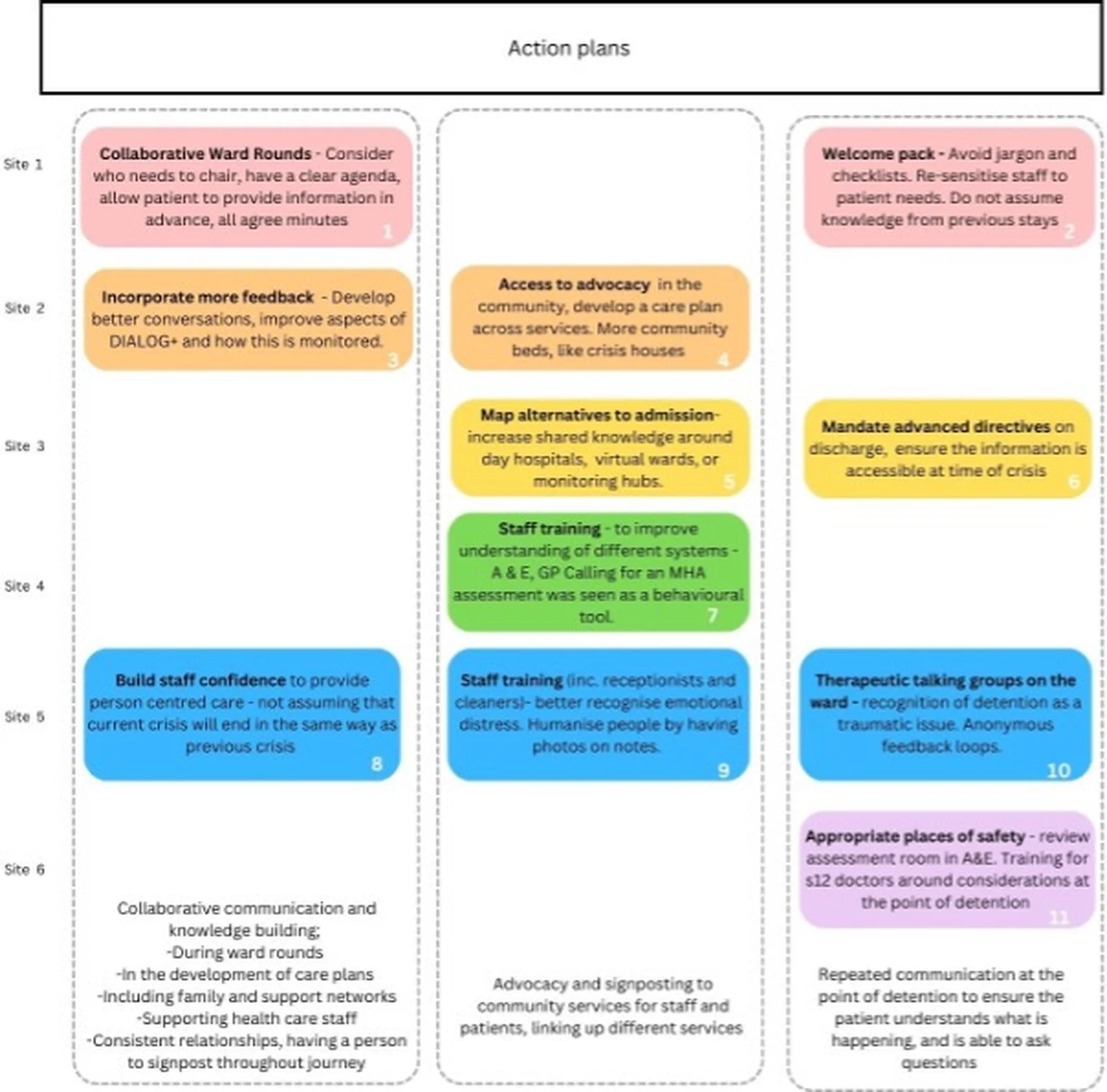
Developed action plans (numbers in boxes index quotes in online supplemental material table 2). A&E, accident and emergency; GP, general practitioner; MHA, Mental Health Act.

### Follow-up at 3–6 months

Despite clear action plans it was challenging for local teams to implement their plans due to local change and committee processes taking time, relationship building, alignment with other local actions and insufficient resources. Indeed many participants fed back how useful they found the images from the photovoice phase of the work to foreground lived experience in the co-design meetings and could envisage various applications of the images in their local systems.

There was some progress in local systems that was shared during the 3–6-month follow-up. Following the co-design workshops, local PIs had limited capacity to champion consequent recommendations; therefore, it was important to identify strategic leaders to take this on and ensure that the information is shared in the relevant spaces where it can have the most impact. Since the final co-design workshops, outputs and policy recommendations have been shared at the following meetings to embed consequent actions into existing groups in the NHS Trusts;

- Bradford—Clinical Board, Mental Health Legislation Committee.
- Derby—Reducing Health Inequalities Delivery Group, s136 Implementation Group.
- Oxford—Mental Health and Law Committee.
- London—Improving Quality of Life Outcomes Steering Group, Community Mental Health Transformation Board.
- Birmingham—Reducing Restrictive Practices Working Group.
- Leeds—Synergi Network Steering Committee.

In addition to the action plans, there have been individual impacts, allied mental health professionals amending their own individual practice to consider the impact of where they conduct Mental Health Act (MHA) assessments and the setting up of a group in Birmingham to (re)consider concepts around Medical Gaslighting in mental health and how to address this concept. There are also ongoing conversations around how some of the materials developed from the photovoice can be used in staff training and inductions that began in the co-design meetings.

There are five national recommendations from this work that evolved from a knowledge exchange event where the local action plans and progress over 6 months were presented;

1. Better connections between local services to improve early access to appropriate care and prevent detentions.
2. Building capacity both in terms of the number of staff available and the skill set of staff to better facilitate services to respond in a timely way.
3. Further co-designing of new trauma-informed standards of care for detained inpatients recognising the traumatic nature of receiving CAT.
4. Providing therapy before, during and after detention.
5. Personalising discharge and community support plans

These could be considered as *Co-PACT quality improvement standards* that sites could adopt and be benchmarked against, particularly in the context of national initiatives to address racial inequalities such as the Patient Carer Race Equality Framework.^17^

## Discussion

### Summary of outputs

#### Connect and grow networks of support

Touchpoints were identified across both groups where patients had sought help but not been able to access it, fuelled by an inconsistent awareness of key actors and which services were appropriate at certain stages of a person’s mental health journey. During workshops, it was discussed that CAT rates could be decreased by better facilitating access to care through better signposting, training for first responders and awareness of any alternatives to CAT in the community such as crisis houses. A systemic review found crisis resolution teams to be effective, but limited evidence of models regarding access to care when in crisis.^18^ Development and implementation of crisis-related mental health systems need to be grounded in the experience rather than dictated by policy. For example, a lack of resourcing has been identified as a barrier to consistent and sustained implementation despite policy mandates.^19^

Improved understanding of local resources beyond mental health services will not only provide benefit in aiding access to care but also in supporting people once discharged to avoid redetention. A scoping review found evidence for the effectiveness in reducing repeated detentions from the use of social interventions, particularly around accommodation, employment and family psychoeducation for people with severe mental illness.^20^

#### Co-design new standards for inpatient care

Staff often reported challenges in reconciling their moral compass with their legal compass, what they wanted to do compared with what they should do. Patients reported challenging interactions with staff, exacerbated by the disorientating environment and dehumanising processes that reduce patients to a diagnosis or set of symptoms.

The process of being detained, where people were held when awaiting assessment and what constitutes a place of safety were all recognised as important touchpoints. Within the photovoice data, an experience had been shared of a patient calling the police from an inpatient unit because they did not feel safe. There is evidence that despite complex differences in perspectives regarding incidences such as patient aggression, mutual understanding can be achieved by developing communication with patients, staff and informal caregivers.^21^ Shared understanding and change in workplace culture could also improve the ward environment. Feeling safe has been recognised as a critical component of a therapeutic environment.^22^ Improved communication, compassion-focused and person-centred training were all proposed as staff training that may improve the environment. A systematic review found limited evidence to support interventions that improve the social climate in mental health units.^23^ There is a need for better evaluation of how interventions are implemented that incorporates continued adaptation and sustained resources.

#### Provide better access to talking therapies

Stories were shared of patients receiving CAT while on the waiting list for therapeutic interventions.

Detention under the MHA was repeatedly recognised by all stakeholders as a traumatic experience for all involved. Adjustment to life postdischarge was also recognised as a time when patients experienced stigma and challenges with their identity thus needed support, yet were often put on wait lists for weeks or months to receive therapeutic support.

In our work, it was the first time for many participants that they had had the chance to discuss their experience with other people who had experienced CAT. There is a growing body of evidence for the benefits of peer support interventions in this population, such as increasing advocacy and social capital.^20^

#### Personalise

The need for personalised advanced planning during discharge conversations was often shared. Patients told stories where a lack of support led to redetention or where their wishes were not followed. More positively, during co-design meetings, there were also experiences shared where people had identified family members and shared a plan in advance of relapsing, which led to CAT not being required.

There have been multiple reviews around the benefit of advanced choice documents in different psychiatric contexts.^24 25^ Despite willingness, barriers to implementation were often cited as a lack of familiarity and confidence from both healthcare professionals^25^ and patients.^24^

#### Family and support

One of the recommendations for the MHA, published in the draft bill in January 2022, was the role of the nearest relative, proposed to be reframed as a nominated person. In this research families and friends were keen to provide support but encountered challenges in accessing information or being acknowledged when someone was detained, which is reflected in existing research with carers.^26^ Research has highlighted that while family members often feel a sense of duty to provide support, there is often a sense of relief when someone is detained.^27^ Family and friends often play a crucial role in a person’s sustained recovery; we need to better support and facilitate collaboration between healthcare teams and patient’s support systems.

### Contextualising our outputs

At the time of collecting the data a draft bill was published in June 2022, the joint committee’s report which contained 55 recommendations was published in January 2023 and the government responded to this in March 2024. We have mapped how our co-designed recommendations relate to the MHA reform (online supplemental material table 3).

From our five recommendations, the co-design of improved standards of care for inpatient units needs to be prioritised. While co-designed outputs should by design and by virtue of engaging multiple perspectives and stakeholders be implementable, there are many challenges in bridging the gap between knowledge and action. Implementation science frameworks may address this by better identifying the evidence—practice gap and ensuring mechanisms by which to both evaluate and adapt implementation of co-designed outputs.^28^ This challenge is compounded by the variation in context and readiness to engage in conversations around inequalities and race as well as competing initiatives in local sites. In future co-design research, at a local level, readiness for implementation should be evaluated prior to undertaking co-design work to better understand the challenges (such as resourcing) that will need to be addressed in the co-design process that pertain specifically to the local context and implementation challenges. National initiatives such as the Race and Health Observatory Implementation Model (https://www.rcpsych.ac.uk/improving-care/nccmh/service-design-and-development/nhs-race-health-observatory-implementation-project) and the Patient Carer Race Equality Framework^17^ provide helpful opportunities to leverage change. Our recommendations could be used at local site levels as quality improvement initiatives and care standards to facilitate meeting the objectives of these national initiatives.

### Strengths

For many people in the first co-design meeting this was the first time that the group were in the room together. There were varying levels of experience in terms of healthcare professionals working with lived experience groups and people with lived experience working alongside healthcare professionals. It was important at the beginning of the workshop to have an ice breaker question that allowed people to introduce themselves as humans with shared experience rather than as their ‘roles’. This also organically allowed for several people who had been brought into the room as healthcare professionals to disclose personal lived experience of mental illness or being informal carers for loved ones detained under the MHA. One of the strengths of this work was curating creative spaces that allowed a diverse group of participants to engage in conversations around difficult experiences in a productive way.

While many people were already aware of the challenges surrounding the experience of being detained and ethnic disparities anecdotally, the use of image data allowed for them to engage with different perspectives and contribute their own experiences. Working in six different local systems highlighted the impact of place on not only the implementation of any action plans but also on the process of convening the co-design workshops. These experiences raise important questions when considering the implementation of local action plans to address national concerns.

### Limitations

Recruitment was challenging. This study was conducted between February 2021 and March 2023 when many were experiencing or recovering from the COVID-19 pandemic and many hospitals were contending with the junior doctors strikes. This meant that capacity in the research and delivery teams as well as senior leaders varied greatly, and often people were unavailable at short notice. This meant that some groups were not well represented at some of the co-design meetings. To mitigate this the local action plans were reviewed and iterated after meetings with the relevant missing stakeholders. The ambition of the research team was for the local NHS trust to have ownership of the resulting action plans and their implementation, so there was not capacity to support this beyond the 3–6-month follow-up.

The scope of this study did not extend to the support and monitoring of implementing the co-designed action plan. Efforts have been made to encourage local working groups or clinical boards in each of the trusts to adopt and monitor the action plans within existing remits and processes. Future work should draw from Normalisation Process Theory (NPT) to better understand and define the context in which an intervention is being implemented.^29^ NPT analysis frameworks are a useful tool to assess whether an intervention is likely to be normalised and to improve complex interventions taking account of the broader system rather than focusing solely on individual behaviours, capabilities and motivations.^29^ Indeed, it is possible that in this research the burden of responsibility on the individuals to implement proposed interventions after the co-design workshops was too great.

We did not have a formal implementation phase in the scope of this project but relied on knowledge diffusion and uptake by individual participants. Establishing timeframes and ascertaining accountability for actions beyond the scope of the project was extremely challenging; there was a preference to default to existing initiatives. The theoretical domains framework^30^ provides a useful guide for bridging the gap between research and implementation by targeting specific behaviours, particularly for systematic intervention design, by drawing from behaviour change theories. This framework could be used to further evaluate and consider why reliance on uptake by individual participants in this context did not work.

### Methodological advances

We innovated the established method of EBCD by centralising lived experience elicited through photovoice workshops. The lived experience data represented the views of both healthcare staff and patients, which were well aligned. Presenting data from both patients (the majority of whom were from a racialised background) and healthcare professionals was useful in addressing power imbalances during the workshops and facilitating diverse groups of people to contribute meaningfully and authentically. We were successful at identifying important touchpoints which might be seen as preventive opportunities that expose particular mechanisms which have not previously been targeted.

## Conclusion

There is a need for collaborative action to address ethnic inequalities in the implementation of the MHA. This research brought together people in six different NHS trusts to review photovoice data gathered from both service users and staff and use it to co-design action plans. The local challenges faced and the diversity among minority populations need to be better considered when considering national policy to effect sustainable change. This work was presented to multiple relevant parties during the process of reforming the MHA, including in the House of Lords. Further work should refine systems interventions that address inequalities and evaluate their implementation in the context of the reformed MHA (2025) using pilot programmes, locally and nationally.

## Supplementary material

10.1136/bmjment-2026-302715 online supplemental file 1

## Data Availability

Data are available upon reasonable request.
