# Supplementary material for: Experience-based co-design of interventions to improve care quality and reduce detention under the Mental Health Act
Source: BMJ Ment Health. 2026 Nov 4;29(1):e302715. doi: 10.1136/bmjment-2026-302715 (PMC13735352; doi:10.1136/bmjment-2026-302715)
Supplement: Supplementary file 1 [file bmjment-29-1-s001.docx]

**Supplementary Table 1: Key points from conversations to illustrate touch points set out in Figure 1**

| 1 | - Medical notes as replacing the person and story: “I don’t know. I don’t know. But like, there’s been all the truth that’s in… The notes, but that becomes the story”. |
| --- | --- |
| 2 | - Psychiatrist on ward round viewed as invalidating experience by the nature of questions was experienced as abrupt. - What is the function of the ward round? – There were queries around whether is it for medication, asking for leave etc. or recovery focused - The ward round was discussed in terms of communication, structure and dynamics following service user and psychiatrist narratives - It was considered whether it would be beneficial to include the focus on recovery in ward round information. |
| 3 | - You contact the GP: “they can't prescribe anything. Erm, you just go round in a circle and by the time the crisis team came, there'd been a week and nobody could come and I couldn't get my prescription for any sleeping tablets to try and help me sleep” |
| 4 | - “I think because they assume that you know everything because you have been there before, whereas actually a lot of times things have changed or its just its less welcoming because its like ohhhh, you’ve been here before, you know everything and then you just left in the room”. - “Actually, it’s a lot of really simple things that could make a difference, but you don’t realise because you’re so in like this is what always happens. You’re not, you don’t know.” |
| 5 | - “…Human being, we're not just symptoms. We're not just medical repositories of medication and restraint and all this nonsense. We don't have to have all of that” - “We can have a conversation.” - There was reference to the Black man as a ‘joke’ stereotype – shuffling, on highest medication when detained. Statistics were referred to that suggest that higher doses of medication are given to a black people on detention. - Stretched (capacity) environment leads to distressed environment so any changes to communication or empowerment were suggested as unable to work in this environment |
| 6 | - Wards were experienced as dehumanizing and staff self- protecting - Discharge was described as an uncaring system. Patients who had been detained for a long time sometimes shared that they forget how to shop, budget, cook. They felt like they were “Really floundering” post discharge to the community. - Mental health is not the same is physical health recovery– different and longer-term systems are needed. - “It's a culture that's existing (not talking to patients as persons)…you don’t have to follow the crowd” |
| 7 | - “It’s like you’re trying to go fishing with the net with really big holes in it and so you lose a lot of people” - On the topic of funding and provision issues that focus on the new thing in a stretched system: “And people coming in to save the world and they just shift all the resources and every time there's a new service that looks like a nice job, all the community teams and the ward band sixes seem to apply for the shiny new service because they're so burnt out”. |
| 8 | - Interactions should be people driven rather than service driven, is a care plan just there to establish what the risk is to the service and how to minimise this risk and keep that person safe or is the care plan about how this person wants to be treated. |
| 9 | - “And get sectioned. You know what? I can’t see a way out, I can’t see a way out, but do you know what. When it happens to early intervention patients there’s more than more chance of them coming back from that”. |
| 10 | - “Think about kind of I guess, how it’s institutional racism is perpetuated in different spaces and how we can foreground that and address it.” - “It’s not patient focussed because you can’t physically do it, so that communication is there, but it’s not of the quality that’s required because of the staff constraints” |
| 11 | - “The need for medical notes to reflect the whole person not just diagnosis” - “But The thing is, if you don't have psychosis, then I don't think you get the same level of care” |
| 12 | - “I’m saying that just about like like prevention so like. I'm saying that just about like like prevention so like. For me, like if I can see myself getting worse before I reach my worst point. I can say to someone that I'm noticing myself getting mad. You know I need help. To be told you're not ill enough.” - On the topic of the patient journey. “So we found that some people identified that if they were on a caseload, for example, they weren't eligible to other services”. - “well, I was just going to say that like, I feel like the communication between services is like quite it's quite poor in the sense of if you go to one service and they signpost you to a different service”. |
| 13 | - “If we were to focus on one thing it would be staff interactions because from that comes coercion” - Debrief and peer support viewed as important after detention to manage what may be experienced as trauma: “And and seeing that you know so many people come out the other end, the experience of the ward is people with PTSD, and I know people have PTSD purely from them or experience.” |
| 14 | - “I think sometimes I'll get back to that issue of communication. We're kind of second guessing way too far down the line and it feels like with people's experience in seeing as human beings, they always think that I'm a I'm an electronic record.” |
| 15 | - “The Mental Health Act assessment in itself is a trauma for your patients and why patient experience everything we've done here, send it out to Staff, they just so they get that awareness and then figuring out together how can it's it's about us making it less trauma, less traumatic, how can we do that?” - “You know, like when people come in that we have a little book to talk to them about what it is, because when they're so fragmented, it's really hard to remember.” - “Has the risk assessment being done with their ligature on a blanket? No. Offer them a blanket, are they? Looking more comfortable. Have they been there for four hours? They should now be offered mattress” |

**Supplementary Table 2: Key points from conversations to illustrate the discussion around the implementation of action plans set out out in Figure 4**

|  | Pathways to sustainable implementation | Barriers to implementation |
| --- | --- | --- |
| 1 | - Piloting it on one ward and evaluating it. Then increase engagement. - Employ Experts by Experience or Peer Support workers to do inductions | - Staff feeling disempowered/burnout - Understaffing and bank staff - High patient turnover |
| 2 | - Co-design a simple training package to allow other people to chair a ward round - Collaborative record of what happened facilitated by the chair - Have a clear agenda that service users can contribute to to set the agenda for the ward round in advance | - Capacity to help service users prepare for ward rounds |
| 3 | - The key purpose (of DIALOG+) is changing the dynamic of the conversation. Isn't that's the question we are trying to solve and co-produce, care, plan and empower the person. | - Understanding and adding to the DIALOG+ system that is already in place, recognising where there are constraints and where there is flexibility |
| 4 | - Window of opportunity pre section which may have an effect on experience. - Capacity framed as a building block for other changes. - Regularly review care plans, that involve multiple organisations pre and post detention. | - Lack of beds for when people do need sectioning, results in a challenge in building trust in the system that help is there when needed - Staffing and involving people from across the system |
| 5 | - Better communication between services, and consider alternatives like safe haven or true colours - Day hospitals/Virtual wards – how can we support people to be well in the community? | - Professions in the community pushed into general roles - High staff turnover – high living costs, no Oxford weighting - Funding |
| 6 | - Advance directives in the community – mandate for when people leaving hospital, ensure the information is meaningful and relevant to the individual - Social worker on ward | - Fragmented staffing - Need for a dedicated role |
| 7 | - Staff training in a collaborative form that incorporates different systems, i.e. liaison psychiatry, and A&E staff and GP and Bed manager. | - Conflicting priorities and perceptions from different parts of the system |
| 8 | - Engage service users to provide meaningful feedback and case study scenarios | - Emotional state of staff – close down ability to be compassionate – who supports them? |
| 9 | - Emotional intelligence for staff alongside technical skills (Compassion-focused training, CFT) - Invite professionals to Derby Wellbeing Collaborative. Monthly for 2 hours | - Volume of work - Professionals may not know how to help - Very prescriptive – diagnoses? Black and white thinking isn’t helpful |
| 10 | - Group meetings on the ward - Debrief following detention/restraint/discharge - Have a director of lived experience in the Trust | - People may worry about the implications of providing honest feedback – coercive system |
| 11 | - Change the space made available for A & E Mental Health Act Assessments at MRI. The room is small and not fit for the task - Include clear information for people whilst waiting for an assessment | - Risk assessment considerations if adding things to the room. - Maintenance of the room - Ensuring information is clear and appropriate might take time to design |

**Supplementary Table 3:** Mapping our recommendations on to the current draft bill issues. The first two columns are quoted from the community care website (https://www.communitycare.co.uk/2024/03/21/mental-health-act-reform-where-the-government-stands/ ; accessed on 8^th^ May 2024)

| Committee Response to Draft Bill | Government Response | Data from Co-Design Workshop | Co-PACT quality improvement standard |
| --- | --- | --- | --- |
| The draft bill would tighten criteria for CTOs; however, the committee went further and recommended they be abolished for part 2 (civil) patients. | The government did not accept these recommendations. It argued that, for some patients, CTOs provided the least restrictive option. It added that the abolition of CTOs would lead to an increase in delayed discharges and over-reliance on other areas of the MHA that were not designed for longer-term cases, such as section 17 leave. | People questioned what the function of the inpatient unit was – whether it was a community asset – a holding space when people felt unsafe to then go and recover in the community when safe to. Others felt that inpatient units should have a therapeutic function. | Clarity around the purpose and resources of different elements of the system would inform the utility and appropriate use of CTOs. Our first and second recommendations around improving access to care in the community and co-designing a new standard of care could facilitate this. |
| The committee recommended that the draft Bill be amended to provide a pilot scheme for patients to appeal treatment decisions at the mental health tribunal. | The government disagreed, arguing that it would not be appropriate for tribunals to be able to make determinations about whether an individual clinical judgement about treatment, made in good faith, is right or wrong in a particular case. | Many sites discussed the procedures around ward rounds (which included treatment decisions) and how these should be amended to ensure that patients and their families/carers are able to meaningfully contribute to treatment decisions. | The process around both how decisions are made and the documentation of these should be more transparent, ward rounds and better engagement of a patient’s support system could facilitate this. Our final recommendation around personalising care and meaningfully engaging families/carers would support the committee response to provide better access to tribunals and more accountability by involving friends and family. |
| The committee recommended that once capacity has been built up in the advocacy sector, the “opt out” scheme should be extended to voluntary patients. It also recommended a central advocacy service, to address shortages in specialist advocacy, and a statutory right to request “culturally appropriate advocacy”. | The government rejected these recommendations. It argued that the draft bill would already extend the right to an IMHA to informal patients and the new opt out scheme should be prioritised for formal patients who are potentially more vulnerable compared to informal patients. | Opt out advocacy was supported particularly to improve access for raciliased populations. Challenges in capacity amongst care co-ordinators was discussed, in the content of the importance of continuity of care (which may increase the demand for an IMHA). There were challenges in accessing mental health support prior to detention, and the possibility of having advocates in the community was considered important, as well as whilst detained and once discharged. | Access to, resourcing and standards for an opt out advocacy system are aligned to our second recommendation around co-designing improved standards of care. |
| The committee raised concerns about a “gap” in mental health care when individuals experiencing a crisis arrived in A&E departments, yet there was no power to formally hold them.  It recommended that the government consult further on a short-term emergency detention power. | The government accepted that there may be a need to provide greater legal clarity to clinicians in A&E and said it would continue to engage with stakeholders to understand how the current legal framework was being applied and what, if any, legislative changes may be required. | It was consistently agreed that A&E’s currently are not an appropriate ‘place of safety’ to hold someone in crisis. One site sought to co-design what the minimum requirements may be for a room in A&E for someone awaiting assessment, similar to the guidance around a s136 suite.  There was also conflicting perspectives around the purpose of an MHA assessment amongst different health care professionals. | This relates to our co-design standard in thinking about what appropriate environments may look like through out the patient journey. |
| The committee recommended a statutory right for detained MHA patients to request an ACD be drawn up. | The government agreed with the aim of this recommendation but argued it was best achieved by placing a duty on services to carry out activity in relation to ACDs, rather than the onus being on the patient to make a request. | The importance of personalisation around ACDs was discussed (and including supports such as family and carers in the process) – as well as challenges around ensuring that these were accessible in a crisis.  It was recognised that ACDs should be part of routine conversations around the discharge process. | This is aligned to the personalisation of discharge plans, and the need for better support upon discharge, people found themselves on waiting lists to access support during this important transition. Discharge plans needs to reflect the individual as well as the resources available to them. |
